# Turning Waste into Sensors: Sustainable Pesticide Detection Using Orange Peel-Derived Laser-Induced Graphene

**DOI:** 10.1021/acsomega.5c07978

**Published:** 2025-10-06

**Authors:** Fabricio A. Santos, Daniel S. Correa

**Affiliations:** Nanotechnology National Laboratory for Agriculture (LNNA), Embrapa Instrumentação, São Carlos, São Paulo 13561-206, Brazil

## Abstract

The growing impacts of climate change, combined with the environmental burden of global pollutants such as agricultural pesticides, have intensified the demand for sustainable and innovative technologies for their monitoring and mitigation. In this study, we introduce a low-cost, easy-to-fabricate, and rapid sensor platform utilizing orange peel (OP)—an abundant agricultural by-product—as a sustainable substrate for the fabrication of laser-induced graphene (LIG). The sensor was designed to detect organophosphate pesticides, namely Malathion and Chlorpyrifos, using electrical impedance spectroscopy (EIS) combined with principal component analysis (PCA) in an electronic tongue sensor array. To improve the structural integrity and reproducibility of the developed sensor, the OP was pretreated with paraffin prior to the laser writing process. Morphological, spectroscopic, and electrical characterizations confirmed the successful formation of conductive graphitic structures. LIG-based devices demonstrated linear sensitivity in the nanomolar range (1 to 20 nmoL^–1^) and retained discriminative capability even in complex matrices, such as tap water. The proposed sensor is simple to fabricate, cost-effective, and enables in situ production using waste biomaterials, offering an accessible and environmentally friendly solution for pesticide monitoring.

## Graphical abstract

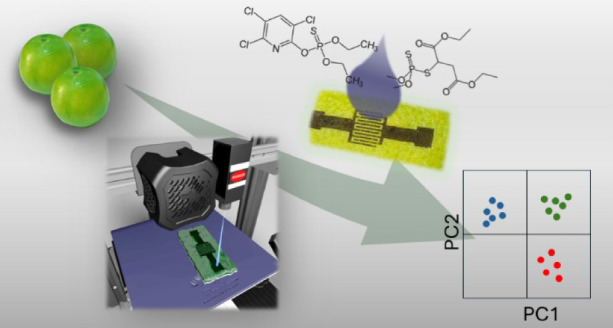

## 1 Introduction

Graphene is a carbon-based material extensively studied in the last two decades due to its remarkable physicochemical properties, such as high electrical conductivity,^1^ large surface area,^2^ and remarkable mechanical strength.^3^ Since its discovery in 2004, various synthesis methods of graphene and graphene-based materials have been explored, including, mechanical exfoliation,^4^ exfoliation by plasma spray,^5^ graphene sheets exfoliated from graphite via electrochemical method,^6^ chemical exfoliation of graphite flakes,^7^ and more recently laser-induced graphene (LIG) technique.^8,9^ LIG is highly promising since it enables the direct fabrication of conductive structures on carbon-rich substrates, such as polymeric matrices (e.g., polyimide (PI)) and natural organic materials, including leaves, wood, fruit peels, and vegetables.^10–12^ The process involves the irradiation of a laser beam, which can operate in different spectral ranges (UV to near-infrared) and modes (continuous, pulsed and ultrashort pulsed), promoting localized pyrolysis and atomic rearrangement of the material’s surface.^13–16^ This results in the formation of porous graphitic structures, with properties distinct from those of the original matrix.^17^ In addition to eliminating the need for toxic reagents and complex processing steps, LIG allows for direct and customizable patterning of conductive elements on a wide variety of surfaces. This versatility makes LIG technique particularly attractive for applications in electrical and electrochemical sensors, wearable devices, and other sustainable technologies.^11,18,19^

Polymeric films are commonly employed as substrates for device production using this technique, with PI being the most extensively used material due to its high carbon content and thermal stability.^9,20^ Other polymers employed for LIG fabrication include poly­(ether imide) (PEI),^21^ polysulfone (PSU),^22^ poly­(ether sulfone) (PES),^23^ sulfonated polyether (ether ketone) (SPEEK)^24^ and polyether (ether ketone) (PEEK).^25^ The efficiency of the graphitization process is intimately linked to the chemical structure of the polymer chain. Polymers rich in aromatic units exhibit a higher trend for conversion into graphitic structures upon laser irradiation, owing to the presence of stable conjugated systems that facilitate the formation of sp^2^ -hybridized carbon networks.^26^ In contrast, polymers predominantly composed of nonaromatic hydrocarbons tend to undergo thermal degradation under similar conditions, leading to carbonization or complete decomposition rather than graphitization.^26^

Although most of the materials currently employed for LIG are derived from nonrenewable petrochemical sources (with their well-known environmental impacts), the use of more sustainable materials is highly keen.^27^ As reported by Bressi et al., approximately one-third of all studies involving LIG are primarily focused on energy storage applications. A wide range of substrates have been employed depending on the target application. For instance, paper sheets, paper filters, nitrocellulose, and lignin are frequently utilized in the fabrication of electrochemical sensors. In contrast, natural leaves, wood, and cork have been extensively investigated for physical sensing platforms, particularly for monitoring parameters such as temperature, humidity, pressure, and mechanical strain.^28^ Thus, we highlight the potential of organic substrates as promising platforms for applications in electrical sensing.

In this context, here we propose a low-cost, waste-minimizing approach using orange peel (OP) as a natural substrate for fabricating LIG-based sensing devices, as illustrated in Figure 1. The use of OP not only reduces reliance on petrochemical-derived materials but also adds value to this abundant agricultural byproduct, since orange is one of the most consumed fruits worldwide.^29^ Naturally rich in carbon and organic compounds, OP offers an ideal matrix for LIG production, enabling the formation of conductive graphitic structures directly on their surface.^30^ Besides, this technique allows for direct writing on the fruit itself (*in situ*), expanding its range of applications. Specifically, the LIG-modified OP substrate was employed for fabricating sensing devices aimed at pesticide detection, which residual presence in food, soil, and water sources poses threat to human health and the environment.^31^ The develop sensors used the concept of electronic tongue (e-tongue), which system is based on a multisensorial system with low selectivity, capable of recognizing and discriminating between different chemical substances in a sample through their electrical, chemical, or electrochemical response profiles.^32–35^ These signals are interpreted using multivariate analysis techniques, such as principal component analysis (PCA) or artificial neural networks (ANNs), enabling both qualitative and quantitative characterization of compounds, even in complex matrices.^36,37^ In this case, the LIG-modified OP devices were integrated into an e-tongue system for the electrical detection of two commonly used organophosphate pesticides, namely Chlorpyrifos and Malathion (Figure 1). Through multivariate analysis of the acquired signals, our approach provides a sensitive, selective, and eco-conscious alternative for monitoring pesticide residues directly on fruit (see Figure S1) or agricultural waste, leveraging the abundance and renewability of these materials. The use of OP combined with paraffin and LIG presents low fabrication complexity, being simpler than many methods that require chemical functionalization, printed electrodes, or lithographic manufacturing processes.^38^

**Figure 1:**
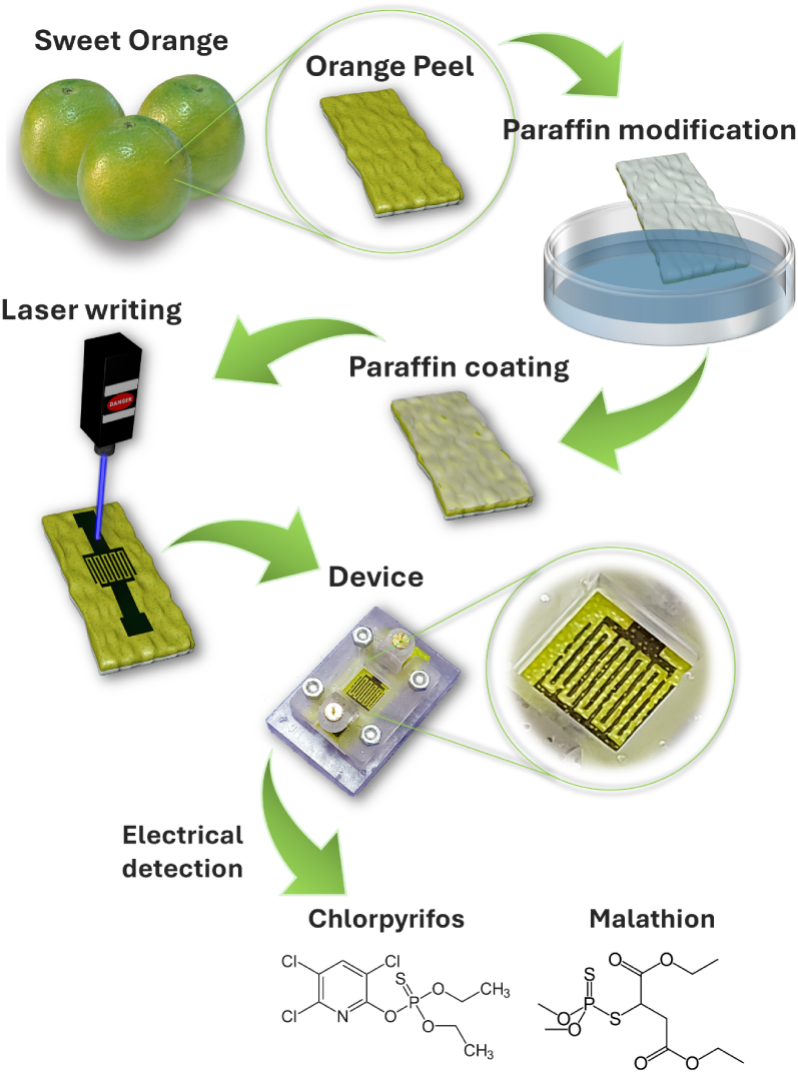
Schematic illustration of the device fabrication process, beginning with the removal of the OP, followed by immersion of the sliced peel in molten paraffin. After solidification of the paraffin layer, laser writing is performed to generate the conductive pattern and complete the device fabrication. The resulting device is then placed into a measurement cell for the EIS analysis of the pesticides Chlorpyrifos and Malathion under different conditions and in various solution media.

## 2 Experimental Section

### 2.1 Chemical and Materials

Monopotassium phosphate (KH_2_PO_4_) and dibasic potassium phosphate (K_2_HPO_4_) were obtained from Sigma-Aldrich. The pesticides Malathion (commercial grade) was obtained from De Sangosse 500 CE, while Chlorpyrifos was obtained from Sigma-Aldrich. Xylene and paraffin were purchased from Êxodo Científica. The stock solutions of each pesticide were prepared and maintained at 4 °C. All the chemicals were used as received, and deionized water was used to prepare all the aqueous solutions.

Pêra Rio Orange (*Citrus sinensis* (L.) Osbeck) were purchased from a local market, washed with detergent, rinsed with deionized water, and dried prior to the experiments. The OP was cut into slices measuring approximately 2 cm × 4 cm and fixed on a support using double-sided tape.

### 2.2 Electrode Fabrication

A 3D-printer (Creality Ender 3 V2, Shenzhen, China) was adapted with a continuous laser operating at 450 ± 5 nm, with a power output of 1.6 W and a beam diameter of 100 μm. The Z-distance was kept constant as 2 mm (focal point), as specified by the manufacturer. The design and engraving on the OP were performed using LightBurn software (v1.6), with a line spacing of 100 μm, variable laser power ranging from 20% to 100%, and scanning speeds from 5 mm·s^– 1^ to 40 mm·s^– 1^. OP measuring 20 mm × 40 mm were cut from the orange fruit. The peel was then briefly immersed in a paraffin solution heated to 60 °C and cooled to room temperature. The LIG process was performed to produce the interdigitated electrodes, consisting of 5 pairs of fingers, with a 500 μm spacing between them. Each finger has a width of 500 μm and a length of 8 mm. The complete electrode design, including all measurements, is provided in the Figure S2.

### 2.3 Characterization

Fourier transform infrared spectroscopy (FTIR, Bruker Vertex 70 instrument) was employed to analyze the OP samples, which were subjected to a prior drying process in an oven at 60 °C for 24 h to ensure complete dehydration. The attenuated total reflectance (ATR mode) were collected in the range from 4,000 cm^–1^ to 400 cm^–1^ with a resolution of 2 cm^–1^ and using 64 scans for each sample.

The morphology of LIG structures was characterized by scanning electron microscopy (SEM, JEOL- 6510), operating at 10 kV. Samples were previously coated with a thin layer of gold using a sputter coater (Leica-SCD 050) before SEM analysis. Digital camera (Xiaomi Redmi Note 13 Pro) was employed to determine the depth profile of OP sample after laser ablation, where images of cross-section were analyzed with ImageJ (National Institutes of Health, USA). Raman spectra were obtained using a LabRAM HR Evolution Raman spectrometer (HORIBA, Japan) using an excitation laser at 473 nm (27 mW) and acquisition time of 10 s. Spectra were collected from 1,000 to 3,000 cm^–1^.

### 2.4 Electrical Impedance Spectroscopy

DC electrical measurements on the electrodes fabricated on OP were performed using a PGSTAT101 potentiostat (Metrohm, Switzerland) in two-electrode mode. AC measurements were carried out using a Solartron 1260 (Solartron Metrology, United Kingdom), with frequencies ranging from 10 Hz to 1 MHz and an amplitude of 50 mV. The samples were measured in phosphate-buffered saline (PBS 1×, pH 7.2) solution and in tap water. A custom-designed support was crafted specifically for this analysis, displaying a well-tailored to accommodate a max solution volume of 600 μL. It was assumed that the moisture content among the different fruits was approximately similar, as all were sourced from the same supplier. To minimize variables that could influence the detection process, electrical measurements were carried out immediately after device fabrication. A delay of several hours or days could result in significant changes in the peel’s properties, potentially compromising the reproducibility of the study.

### 2.5 Analysis

After collecting the EIS data, the results were analyzed using principal component analysis (PCA). This is a statistical technique for dimensionality reduction commonly employed in data analysis. It transforms a high-dimensional data set into a lower-dimensional space by projecting the data onto a new set of orthogonal axes, principal components, that are linear combinations of the original variables.^39^ These components are ordered by the amount of variance they capture from the data, with the first principal component (PC1) accounting for the highest variance, followed by PC2, and so on. By retaining only, the leading components (typically PC1, PC2, and optionally PC3), PCA reduces data complexity while preserving the intrinsic structure and variance distribution of the original data set, facilitating efficient data visualization, noise reduction, and feature extraction.^40^

## 3 Results and Discussion

### 3.1 OP-Based Device Fabrication

Due to its complex chemical composition, media carbon content (42 – 49% w/w), high moisture mass, and the presence of other compounds, OP does not exhibit a very predictable behavior when subjected to laser-induced transformation.^41–43^ In some instances, laser energy led to excessive carbonization of the material, thereby compromising the formation of graphene layers; in others, the energy was insufficient to induce adequate pyrolysis. Besides, issues related to the adhesion and uniformity of the graphene on the peel surface emerged, limiting the performance of the devices intended for sensor applications. Additionally, the inherently irregular and porous nature of the OP surface posed further challenges for the formation of continuous graphene layers, resulting in devices with inconsistent sensitivity and performance. It is worth noting that even for conventional polymeric substrates, delamination of the LIG layers is a problem commonly reported, particularly under conditions of excessive heat generation during the laser processing.^44^

To address this issue, paraffin, commonly used in the fabrication of carbon paste electrodes,^45^ was utilized as a supporting material. Figure 1 illustrates the complete fabrication process of the devices. Initially, slices of OP were cut and briefly immersed in paraffin, preheated to just above its melting point (∼60 °C). Within a few seconds, a thin, solidified paraffin layer formed on the surface of the OP. Subsequently, the samples were subjected to LIG for device writing. After fabrication, the devices were mounted in a custom cell for the detection of Chlorpyrifos and Malathion at various concentrations and in different solution media.

During the graphitization process, the heat generated caused the paraffin to melt, allowing it to infiltrate the carbonized regions of OP. Upon cooling, the paraffin solidified again, forming a thin film that enhanced the structural rigidity of the substrate and contributed to the effective adhesion of the LIG to the OP surface. Figure S3 presents microscopy images that illustrate this effect. In Figure S3a,b­(nonparaffin), a typical coloration of the graphitized regions is observed, whereas in Figure S3c,d­(with paraffin), a bluish hue is noticeable, indicating the presence of paraffin embedded within the graphitized structure.

Image of the fabricated device directly write on the surface of an OP via LIG is shown in Figure 2a. Due to the hydrophilic nature of the LIG produced and the hydrophobic characteristics of the OP substrate, it was necessary to develop a customized support to contain the measurement solution, as depicted in Figure 2b.

**Figure 2:**
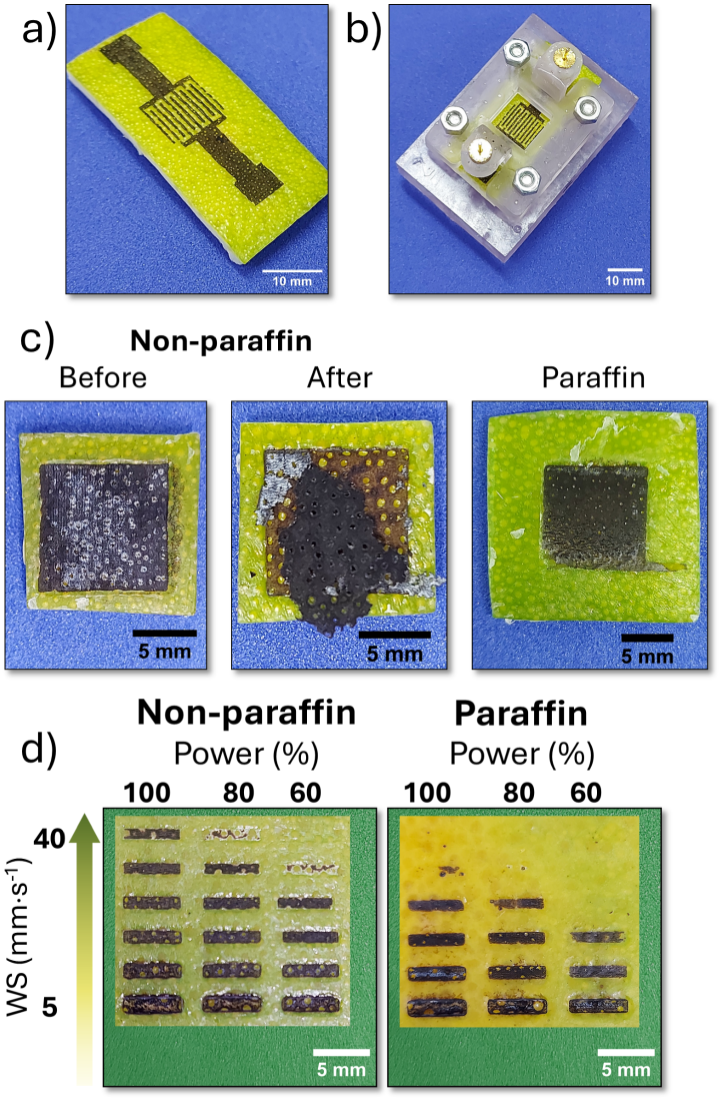
Digital picture of (a) OP slice containing LIG and (b) sample after LIG formation placed in the measurement cell; (c) LIG samples from left to right: uncoated (nonparaffin), immediately after laser writing, and after a period of time showing delamination of the graphitized layer, and a sample coated with paraffin; (d) writing on OP, varying the laser power and writing speed (5, 10, 15, 20, 30, and 40 mm·s^–1^), for samples nonparaffin and with paraffin.

OP, being a complex organic material, exhibits more unpredictable behavior in response to LIG. In some cases, laser energy led to excessive carbonization of the material, hindering the formation of graphene layers, while in others, the energy was insufficient to induce proper pyrolysis.^17^ This is due to the composition of the OP, which consists mainly of two regions: the flavedo and the albedo. The flavedo contains oil glands whose distribution and volume vary from one fruit to another.^29^ Additionally, issues related to the adhesion and uniformity of the graphene on the peel surface are present, limiting the performance of the devices designed for sensor applications. The irregular and porous surfaces of the orange peels made it difficult to form continuous graphene layers, resulting in devices with inconsistent sensitivity and performance. Therefore, we employed an alternative strategy using paraffin (which is widely employed as a support material in carbon paste electrodes)^45^ to modify the OP surface. Figure 2c shows a 10 × 10 mm square written on the surface of an uncoated (nonparaffin), followed by an image of the same region after the delamination of the graphitized layer over time. The next image displays the OP coated with paraffin, followed by LIG. Both experiments were conducted using a laser power of 1.6 W, writing speed of 10 mm·s^– 1^, and two writing scans.

To optimize the graphitization conditions, LIG samples were produced by systematically changing the laser power (60%, 80%, and 100%) corresponding to 0.96 W, 1.28 and 1.6 W, and writing speed (5, 10, 15, 20, 30, and 40 mm·s^–1^) in two scans, for paraffin and nonparaffin samples showed in Figure 2d. Visually, a reduced formation of LIG can be observed during the writing process under higher speeds and lower laser power conditions. Furthermore, in the samples treated with paraffin, an even more limited formation of LIG is evident on the surface of the OP.

### 3.2 OP Characterization

Determination of optimal parameters for device fabrication was conducted by varying the laser power 0.96 W, 1.28 and 1.6 W, the writing speed (5 to 40 mm·s^–1^), and the scans (1 to 8) over the same location scanning. Figure 3a (i – (iv), shows cross-sectional images of OPs subjected to laser writing under two distinct experimental conditions, with and without the application of paraffin. It can be observed that increasing the number of laser scans results in greater penetration depth into the substrate, attributed to energy accumulation and the consequent rise in local temperature. Additionally, the presence of paraffin alters this dynamic, reducing the laser’s penetration capability. Figure 3b illustrates that under low-power conditions, the penetration depth in samples with and without paraffin was relatively similar. However, as the laser power increased, the difference between the two conditions progressively diminished, indicating that the critical ablation energy of the surface layer is more readily achieved in the absence of paraffin. For a number of scan fewer than four, no significant difference in penetration depth was observed, as pyrolysis remained confined to the flavedo region, which is characterized by higher structural density and greater thermal resistance.^46^ Nevertheless, when the number of scans exceeded four, an exponential increase in penetration was observed. This behavior can be attributed to the transition into the inner layers of the substrate, which are predominantly composed of albedo. Albedo contains a higher pectin content (29.79% to 38.21%) compared to the flavedo (20.28% to 29.35%).^47^ Due to its spongy structure and high moisture content (approximately 85%), the albedo exhibits greater water retention capacity, making it consequently more susceptible to ablation during the process.^46^

**Figure 3:**
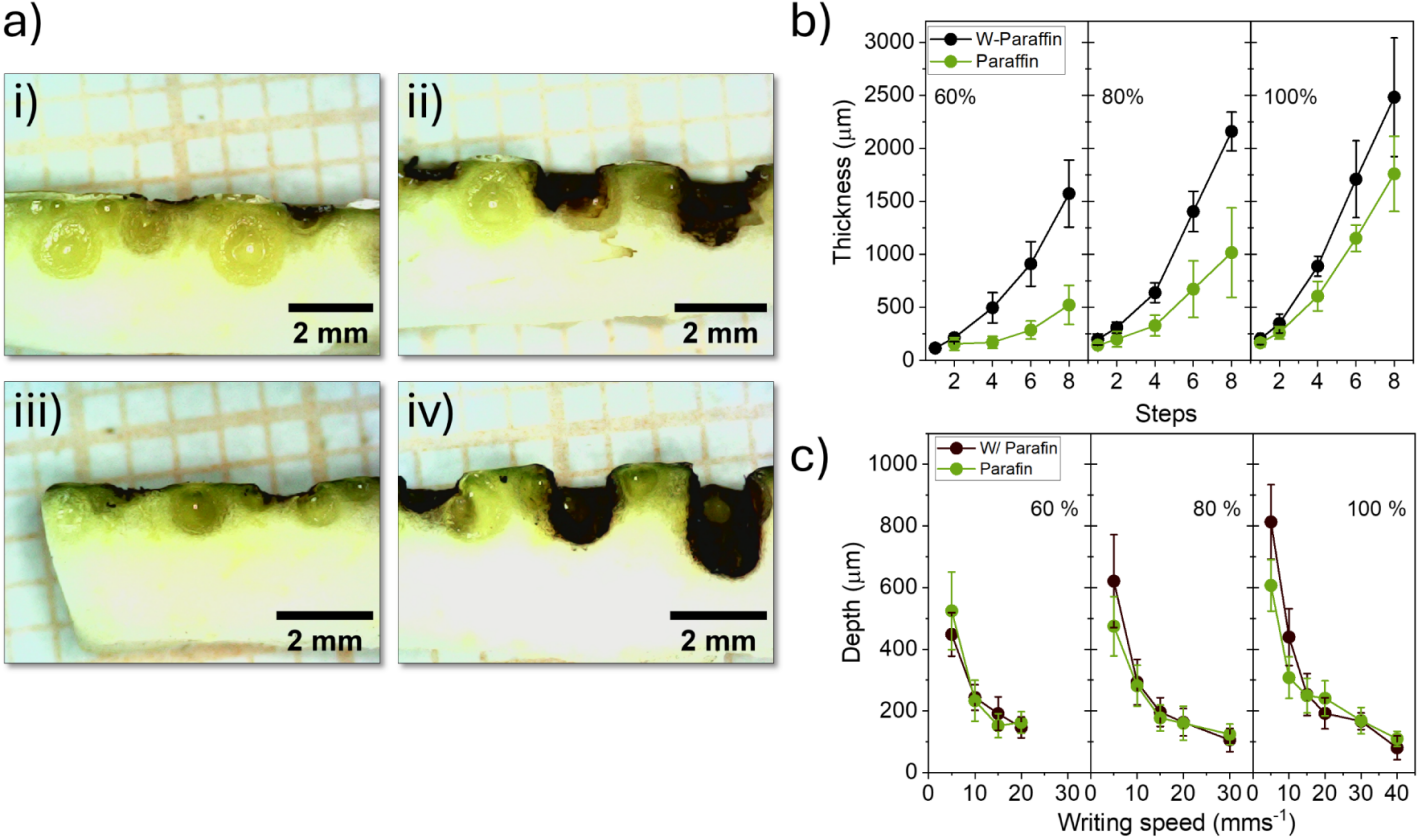
(a) Cross-sectional digital images of LIG samples fabricated on OP at 80% laser power and a scanning speed of 10 mm·s^– 1^: (i) sample without paraffin after scans 1 and 2; (ii) sample without paraffin after scans 4, 6, and 8; (iii) sample with paraffin after scans 1 and 2; (iv) samples with paraffin after scans 4, 6, and 8; (b) laser penetration depth as a function of laser power and the number of scans; (c) laser penetration depth as a function of laser power and laser writing speed.

Conversely, variations in writing speed had no significant effect on the differences of penetration depth between samples with and without paraffin, which remained relatively constant under all evaluated conditions, as shown in Figure 3c. However, regardless of the presence of paraffin, it was observed that the penetration depth decreased exponentially with increasing beam speed. This behavior can be attributed to the reduced interaction time between the laser and the substrate; as the writing speed increases, the fluence decreases, becoming insufficient to promote deep pyrolysis. Additionally, higher writing speeds facilitate faster surface cooling, which further limits thermal propagation into deeper layers of the material.

### 3.3 Spectroscopic and Morphological Characterization

The FTIR results of OP before and after the LIG process, with and without paraffin, are shown in Figure 4a. The bands at 2,964, 2,916, and 2,849 cm^–1^ corresponds to C–H stretching, along with vibrational bending modes at 1,435 and 1,375 cm^– 1^, attributed to aliphatic – CH_2_– and –CH_3_– groups, which constitute the basic structure of the lignocellulosic material in OP.^41^ A significant decrease in the intensity of these bands is observed after laser processing, indicating a strong tendency toward chemical modification during the LIG process.^41^ The vibrational mode at 1,728 cm^– 1^ is associated with C = O carboxylic stretching, and the disappearance of this band for the samples after the LIG process suggests a deoxygenation process of the substrate.^48^ In the region at 1,643 cm^– 1^, a narrow band extending to 1,600 cm^– 1^ is present in the spectrum of the raw peel, while in the LIG samples, this band becomes broader and shifts to 1,570 cm^– 1^ in samples without paraffin and to 1,600 cm^– 1^ in samples with paraffin. These peaks are attributed to aromatic ring vibrations within the lignin structure, indicating structural variations in lignin, the primary component involved in LIG formation.^12^

**Figure 4:**
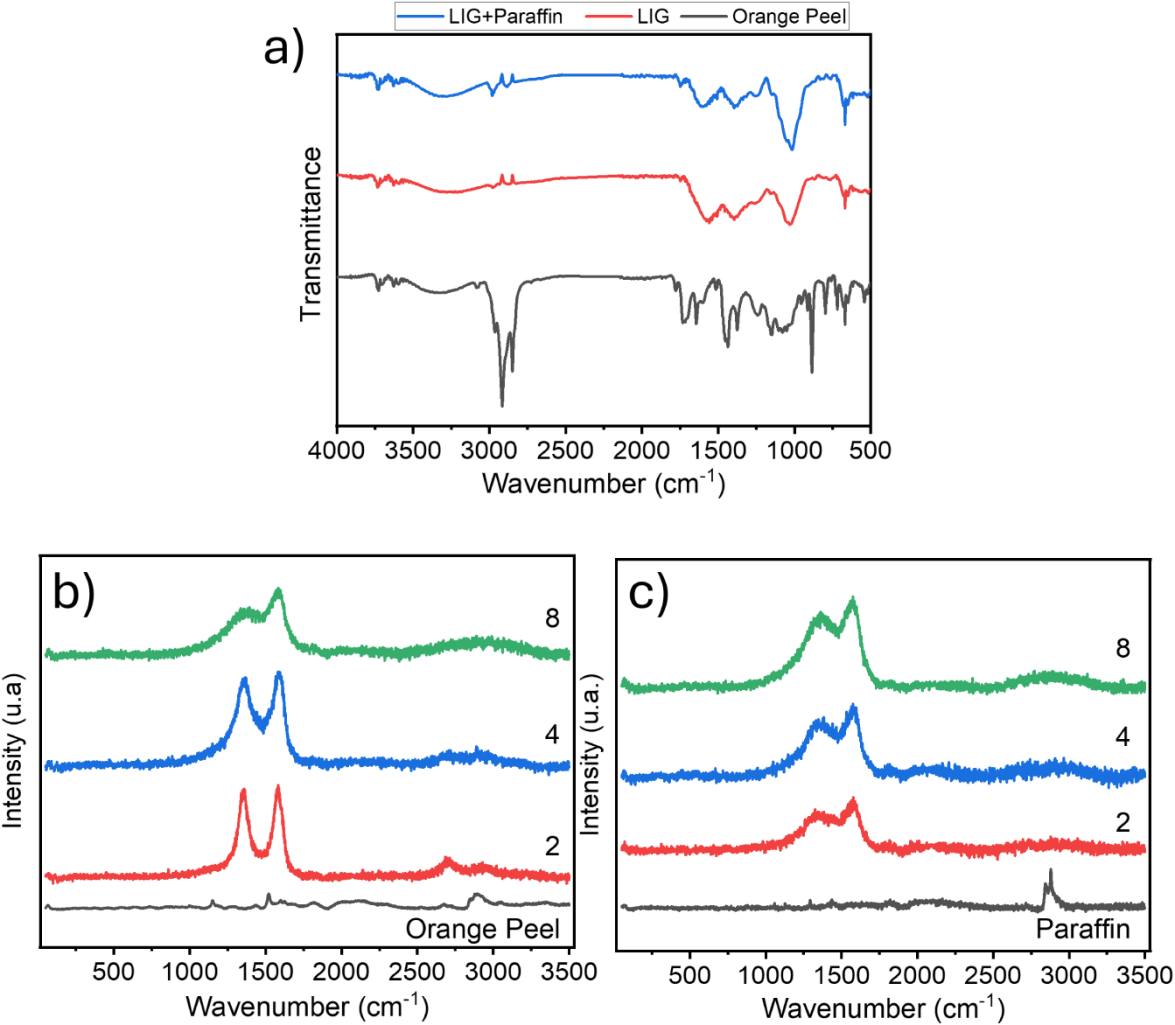
(a) FTIR spectrum of OP before and after the LIG process, with and without paraffin; (b) Raman spectra of samples without paraffin subjected to different laser exposure scans: 2, 4, and 8; (c) Raman spectra of samples with paraffin, along with a reference spectrum of pure paraffin.

Figure 4b,c shows Raman spectra for samples subjected to 2, 4, and 8 laser scan cycles. In the samples without paraffin, it is observed that the longer the laser exposure, the more the D-band intensity decreases, while its width increases, indicating increased heterogeneity in the defect structure of the carbon-based material.^49^ Additionally, the 2D band (∼2,700 cm^– 1^) diminishes in intensity, suggesting the formation of a more amorphous material, which is corroborated by the increase in the FWHM of the G-band, rising from 70 to 107 cm^– 1^, thus indicating a reduction in sp^2^ graphitic domains.^49,50^ This change also reflects in the material’s electrical properties, leading to a decrease in electrical conductivity.^51^ In contrast, the sample containing paraffin exhibits greater stability in the electronic properties of the fabricated material, with minimal changes upon laser exposure. The most notable difference lies in the D-bandwidth, indicating a greater dispersion of defects in the crystalline network.

The morphology of the devices fabricated on OP was analyzed using scanning electron microscopy (SEM). Figure 5 presents a comparison of the peel surfaces after the LIG process without (a–c) and with paraffin (d–f), at different magnifications. In the paraffin-coated samples, a clear surface coating is observed in the areas where LIG was produced. Conversely, in the samples without paraffin, the pores formed during the process are clearly visible. This porous pattern is characteristic of the LIG technique and directly influences the material’s properties.^15^ The presence of paraffin, in turn, contributes to a more uniform surface, minimizing the exposure of these pores, but it can also limit the sensitivity of the system.

**Figure 5:**
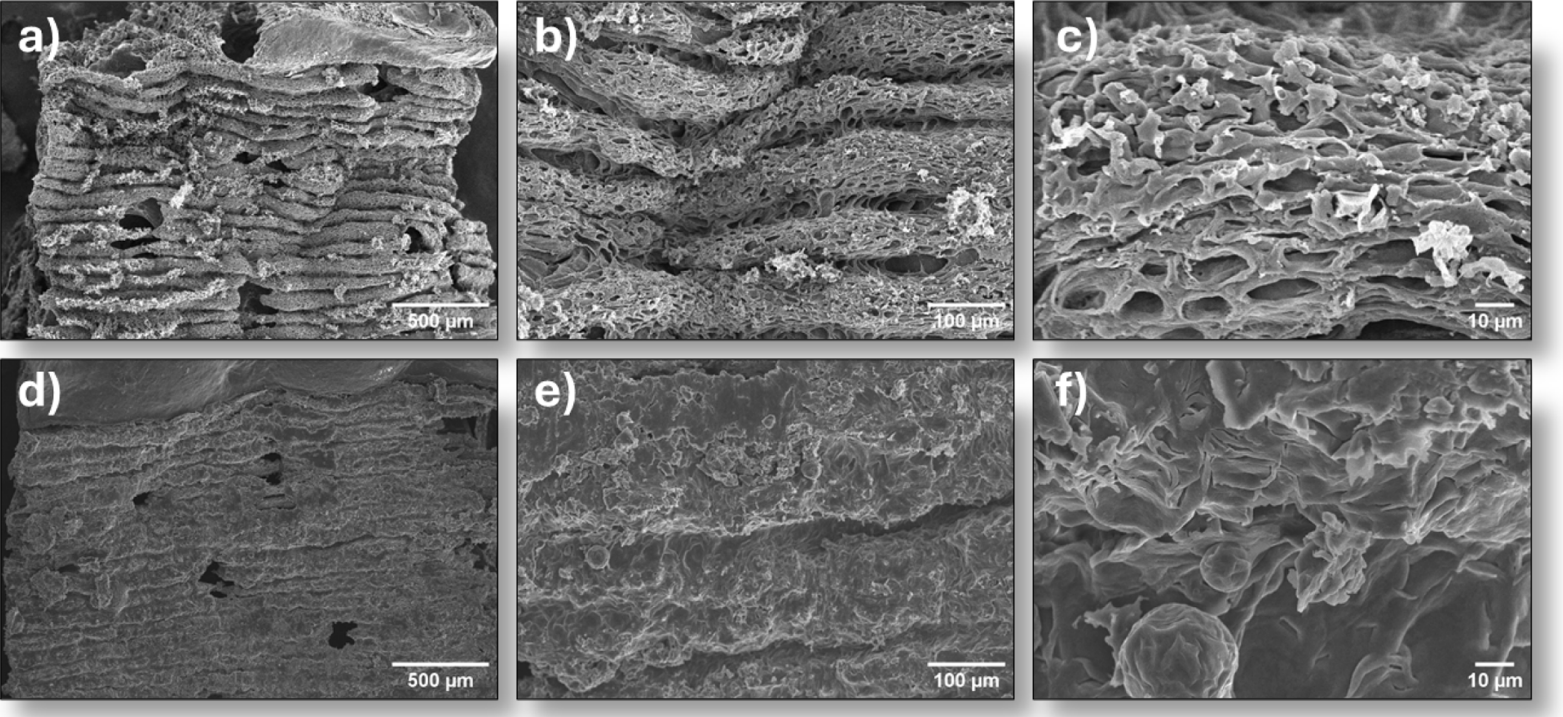
SEM images comparing the morphology of LIG without paraffin at different magnifications: (a) 50×; (b) 200×; (c) 1000×; and with paraffin: (d) 50×; (e) 200×; (f) 1000×.

Figure S4 presents cross sections SEM images of LIG in OP samples with and without paraffin. The images reveal that paraffin infiltrates the pores of the peel during the carbonization process. Paraffin begins to decompose at temperatures above 235 °C,^52^ while the laser-induced carbonization process reaches temperatures exceeding 550 °C.^12^ As a result, part of the paraffin decomposes, producing smaller, volatile hydrocarbon chains. This decomposition is evidenced by gas emissions released from the samples and the formation of a thin film on the laser-exposed surface.

### 3.4 Electrical Characterization

Due to the high porosity of the devices produced via LIG, the conventional four-point probe technique for measuring electrical conductivity proved inadequate (evaluated in subsidiary experiments not shown here), yielding inconsistent data. Consequently, the electrical conductivity of the devices was assessed by two-point resistance measurements, employing fixed electrodes on samples with standardized dimensions of 6 × 12 mm. In this experiment, different processing conditions were investigated by varying the laser power (60%, 80%, and 100%) and the writing speed (5, 10, 15, 20, 25, and 30 mm·s^– 1^), as shown in Figure 6a. It was observed that for samples without paraffin addition, the electrical resistance remained relatively stable across different laser power settings and writing speeds, ranging only between 10 and 20 kΩ for writing speeds from 5 to 25 mm·s^– 1^. This behavior suggests a well-formed graphitic carbon network and a homogeneous distribution of conductive structures across the surface. In contrast, samples containing paraffin exhibited more pronounced variations in electrical resistance, with values ranging from approximately 20 to 200 kΩ for writing speeds between 5 and 15 mm·s^– 1^. This more resistive behavior was expected, as structural analysis indicated the incorporation of paraffin into the carbonized matrix. The presence of paraffin partially disrupts the continuity of the conductive graphene network, introducing insulating barriers that increase the overall device resistance.

**Figure 6:**
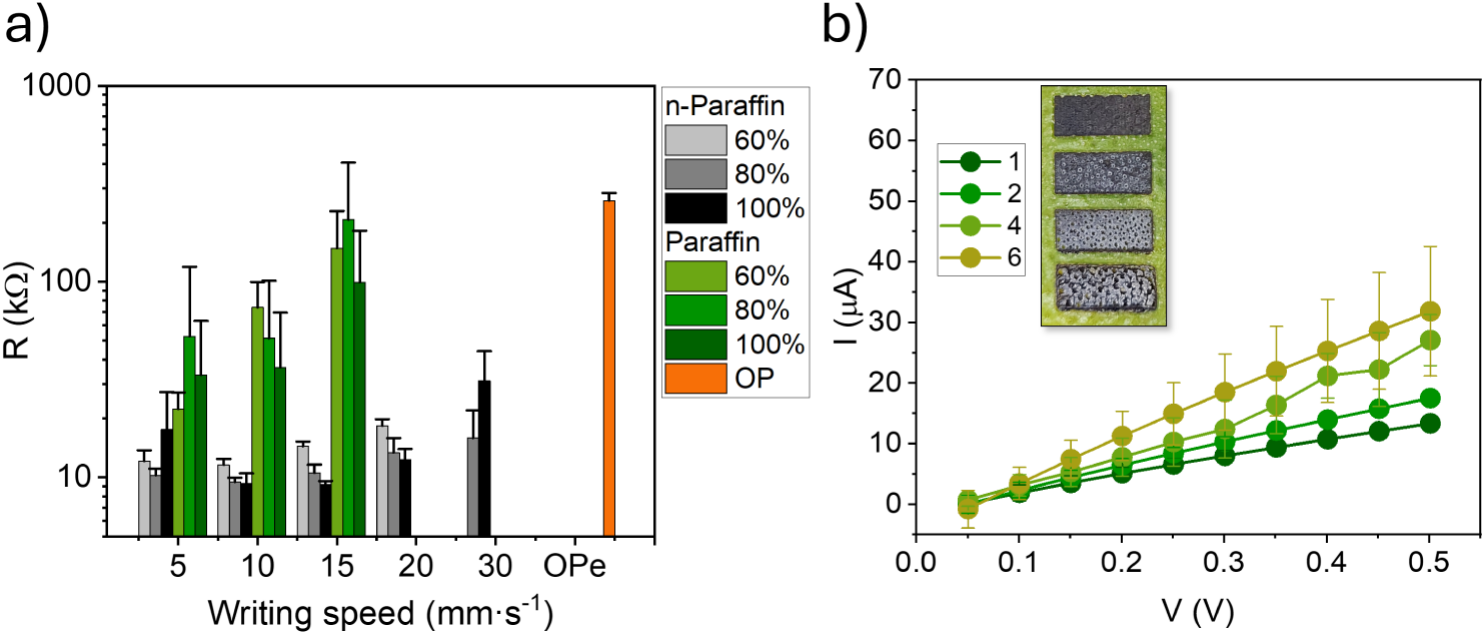
(a) Electrical resistance measurements of LIG produced on OP by varying the laser power and writing speed, with the number of scans fixed at 2, including samples both with and without paraffin, and the electrical resistance of untreated OP. The samples employed measured 6 × 12 mm; (b) current–voltage (I × V) curves representing the average response of nine devices, obtained by varying the number of laser scans. The inset shows the layout of the devices used, with the number of laser scans ranging from 1 to 6.

Figure 6b, presents the I × V curves obtained with varying numbers of laser scans, while keeping the laser power constant at 100% and the writing speed at 10 mm·s^– 1^. Inset shows the layout of the devices used, with the number of laser scans from 1 to 6. For samples subjected to more than two scans, an increase in the standard deviation was observed, reflecting a trend similar to that seen in the laser penetration depth experiments. This trend suggests reduced reproducibility and greater variability in devices exposed to additional scanning cycles, likely due to the excessive energy accumulation and uneven heating across layers. The increased variability can be attributed to thermal effects, which, over successive cycles, result in irregular heating of the layers, compromising the consistency of the results. This phenomenon may adversely affect device performance, making it more prone to failure and reducing its overall efficiency.

Electrical characterization under alternating current (AC) conditions was performed on 30 devices, all fabricated following the previously established protocols (power 1.6 W, writing speed at 10 mm·s^– 1^ and two scans). The design employed for the electrodes is illustrated in Figure S2. Measurements were conducted in phosphate-buffered saline (PBS, 0.1 mol L^– 1^, pH 7.2).

Figure S5a,bpresent the electrical impedance curves and the corresponding Nyquist plots for the 30 fabricated electrodes. The observed variability in the impedance response at 1 kHz was approximately 20%, which is primarily attributed to the inherent heterogeneity of the OP samples. This variability is expected, considering the natural differences in chemical composition, texture, and thickness between peels from different fruits. Such sample-to-sample variations are intrinsic to the use of organic, biological substrates like fruit peels, which inherently exhibit nonuniform characteristics.^53^ Thus, the variability in the electrical properties of the devices can be directly attributed to the intrinsic differences in the material. Although standardized fabrication procedures have been implemented and a measurement system tailored to the physical properties of the substrate has been developed, achieving uniformity across all samples remains a significant challenge. The biological variability of the organic precursor OP is one of the main factors contributing to the dispersion observed in the electrical characterization data.^46,54^

### 3.5 Detection of Malathion and Chlorpyrifos Pesticides Using LIG Sensor in OP

Electrical impedance measurements of the system were performed by sweeping frequencies from 1 MHz down to 10 Hz. However, the ideal response range for this type of sensor lies between 100 Hz and 100 kHz, which is related to the surface coating properties of the electrode and its interaction with the analyte (*f* <100 Hz are dominated by the formation of the electrical double layer, while responses for *f* > 100 kHz are usually associated with the geometric characteristics of the electrode).^55^ Initially, the measurements were conducted in 1× PBS solution with a pH of 7.2, which helped minimize the influence of the medium on the sensor’s response.

As proof of principle, the system was employed to detect the presence of two organophosphate insecticides, namely Chlorpyrifos and Malathion, which are important in citrus farming. The concentrations used were 1, 5, 10, and 20 nmol L^– 1^. In this type of analytical device, variability in response between different units is common, which can compromise the ability to distinguish a positive signal and worsen the detection limit, especially in highly diluted solutions.^56^ Our system, based on waste organic substrates, faces additional challenges due to the intrinsic variability of the material, which may alter its characteristics over time and between samples. To mitigate this variability, we derived the Z’’ curve as a function of Z’ for all measurements. Based on these data, the normalized response as a function of analyte concentration was calculated by subtracting the derivative value of the blank buffer (without analyte) from that of the sample containing the analyte, and then dividing the result by the derivative of the blank buffer, as shown in the equation:

1 $$\frac{{\left(\frac{dZ^{\prime \prime }}{dZ^{\prime }}\right)}_{\mathrm{sample}}-{\left(\frac{dZ^{\prime \prime }}{dZ^{\prime }}\right)}_{\mathrm{blank}}}{{\left(\frac{dZ^{\prime \prime }}{dZ^{\prime }}\right)}_{\mathrm{blank}}}$$

The response obtained is like an electronic tongue system, in which a low-selective recognition element is present. In this way, the system evaluates how different analytes interact with a given electrode, or how multiple electrodes respond to the same analyte. The linear combination of these responses, as a function of one or more variables, is analyzed using clustering algorithms such as PCA.^32^ To discriminate between different analytes, we applied PCA at a frequency of 1 kHz using a set of five devices, in which each point represents the linear combination of the responses from these devices. As shown in Figure 7a, good discrimination was achieved, with a single principal component (PC1) explaining 91.4% of the variance, demonstrating linearity of detection for both pesticides (Figure 7bc).

**Figure 7:**
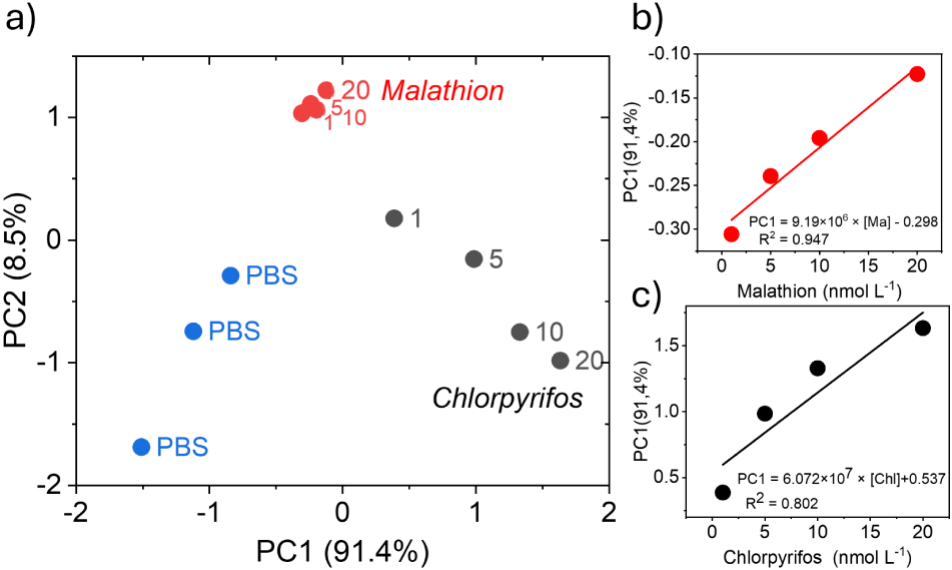
(a) PCA performed using five LIG devices on OP with PBS solution containing Malathion (Ma) and Chlorpyrifos (Chl); (b) PC1 as a function of Malathion concentration in PBS; (c) PC1 as a function of Chlorpyrifos concentration in PBS.

To simulate analysis under real-world conditions, tap water samples were prepared and spiked with the pesticides at the same concentrations used in the buffer solutions, as well as mixtures of the two pesticides in varying proportions de Chlorpyrifos e Malathion respectively: (1 + 20, 5 + 10, 10 + 5, and 20 + 1 nmol L^– 1^). The sensor was able to discriminate all solutions, as shown in Figure 8a. However, under these conditions, a decrease in the contribution of PC1 and an increase in the contribution of PC2 were observed, together accounting for 82% of the total variance.

**Figure 8:**
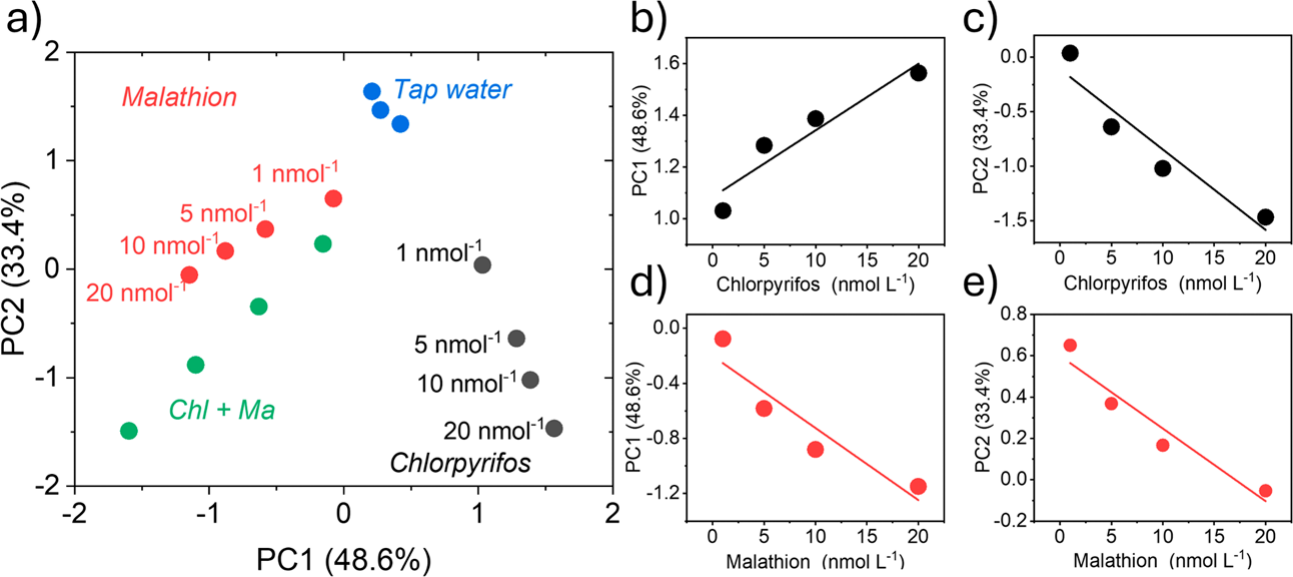
(a) PCA performed using five LIG devices on OP with tap water solution containing Ma and Chl and the sum of Chl + Ma; (b) Linear regression for Chlorpyrifos in PC1 (PC1 = 2.57 × 10^7^ × (Chl­(nM)) + 1.08); (c) Linear regression for Chlorpyrifos in PC2 (PC2 = – 7.39 × 10^7^ × (Chl­(nM)) – 0.11); (d) Linear regression for Malathion in PC1 (PC1 = 1.40 × 10^7^ × (Ma­(nM)) – 0.20); (e) Linear regression for Malathion in PC2 (PC2 = – 3.52 × 10^7^ × (Ma­(nM)) + 0.60).

A reduction in the system’s ability to distinguish the analytes was anticipated, given the increased complexity of the tap water matrix. Additionally, the interaction between the water and the OP substrate may lead to pH variations, which can affect sensor performance. Despite these limitations, the linear detection of the analytes was well represented by the first two principal components, as illustrated in Figure 8b–e. Even with changes in matrix composition, the sensor system demonstrated satisfactory discrimination capabilities, supporting its feasibility as an effective sustainable platform for pesticide detection in real-world environments, which was based on an agricultural waste.

## 4 Conclusion

We demonstrated the feasibility of utilizing orange peel (OP), an abundant and renewable agro-industrial byproduct, as a sustainable substrate for the fabrication of LIG sensors. The addition of paraffin significantly improved the structural integrity and reproducibility of the devices, mitigating challenges associated with the irregular and porous nature of the OP matrix. Comprehensive spectroscopic, morphological, and electrical characterizations confirmed the successful formation of conductive LIG structures on the OP surface. The LIG-based sensors exhibited satisfactory performance for the detection of organophosphate pesticides Malathion and Chlorpyrifos, at nanomolar concentrations using Electrical Impedance Spectroscopy and data processing through PCA. Overall, this study presents an environmentally friendly and low-cost strategy for sensor fabrication, utilizing agricultural waste and straightforward processing techniques. The findings demonstrate the potential of OP-based LIG devices in the development of sustainable sensing platforms for environmental and food safety monitoring. By employing widely accessible tools, such as a modified 3D-printer and a diode laser, the fabrication process becomes feasible even for users with limited technical skills. While this may not represent the most conventional route for pesticide detection, the emphasis on green and accessible technology makes it particularly suitable for decentralized use, especially in rural or low-infrastructure agricultural environments.

## Supplementary Material



## Notes

Photographic image of LIG on an orange peel, design and dimensions of the devices, optical microscopy of the devices, scanning electron microscopy image, electrical impedance of a set of devices (PDF)
