# Supplementary material for: Turning Waste into Sensors: Sustainable Pesticide Detection Using Orange Peel-Derived Laser-Induced Graphene
Source: ACS Omega. 2025 Oct 6;10(41):49126–35. doi: 10.1021/acsomega.5c07978 (PMC12547790; doi:10.1021/acsomega.5c07978)
Supplement: Supplementary file 1 [file ao5c07978_si_001.pdf]

Supporting Information

Turning Waste into Sensors: Sustainable Pesticide Detection Using Orange Peel-Derived Laser-Induced Graphene

Fabricio A. Santos[1, *], Daniel S. Correa[1, *]

[1] Nanotechnology National Laboratory for Agriculture (LNNA), Embrapa Instrumentação, São Carlos, SP, Brazil;

*Corresponding Authors:

fabricioaps@alumni.usp.br *(F.A.S.);*

daniel.correa@embrapa.br *(D.S.C),*

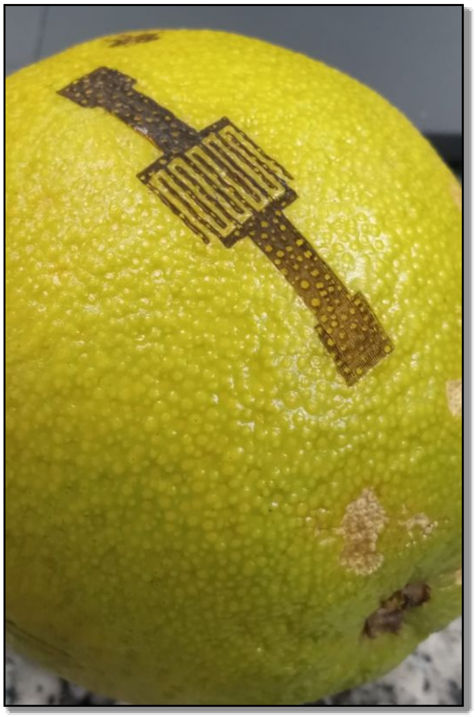

Figure S1: Digital picture of LIG electrode written directly on the orange fruit.

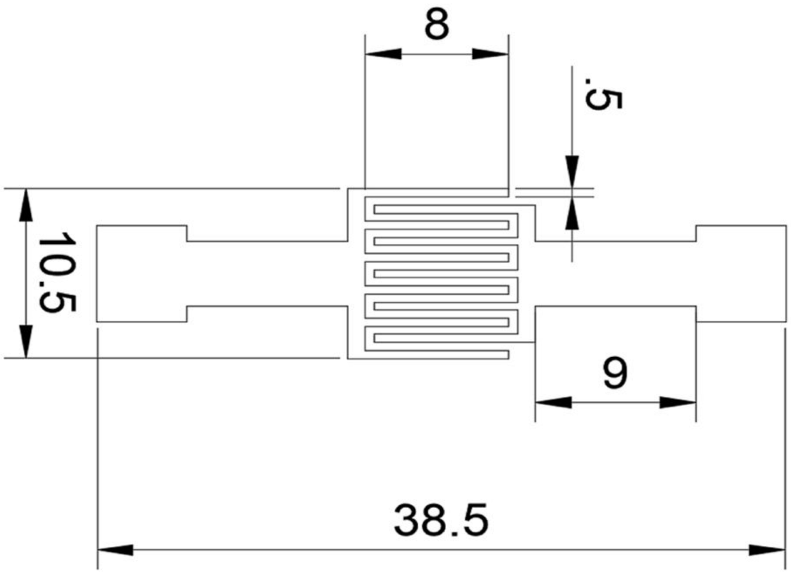

Figure S2: Design of the interdigitated electrode. Scale in millimeters.

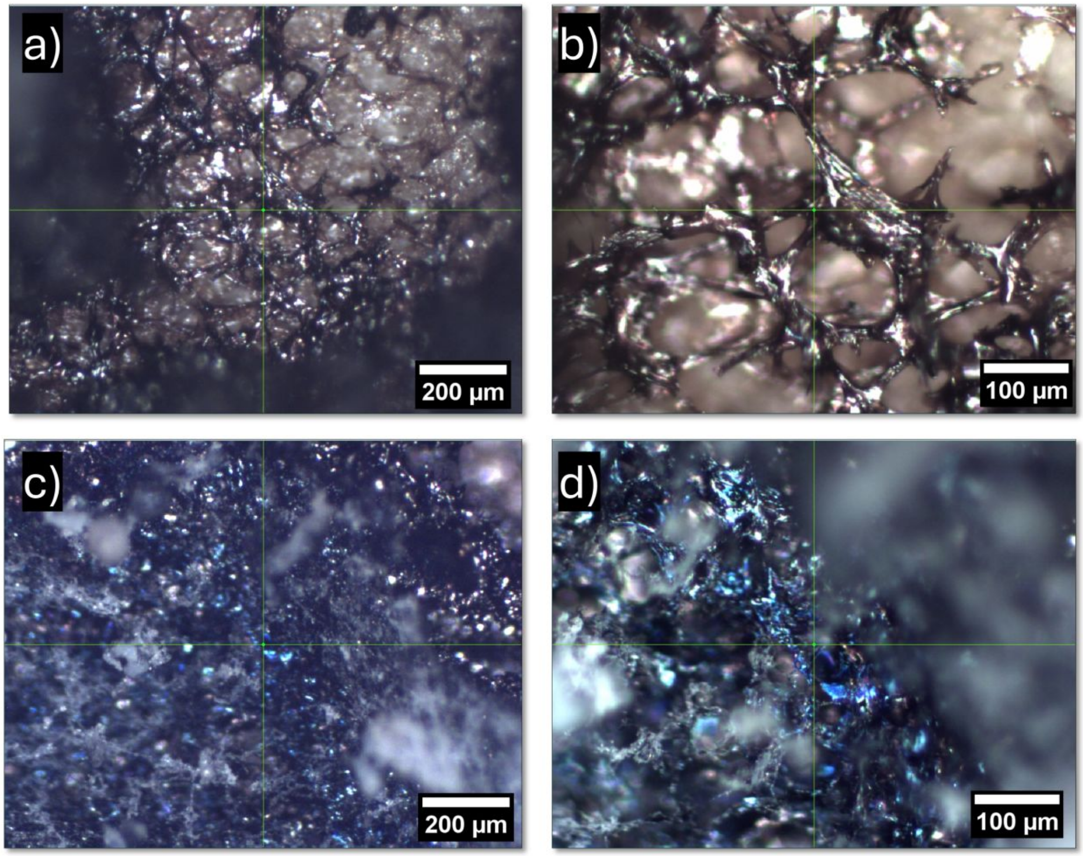

Figure S3: Optical microscopy images (acquired at 5× and 10× magnifications) of a) LIG sample without paraffin; b) LIG sample with paraffin.

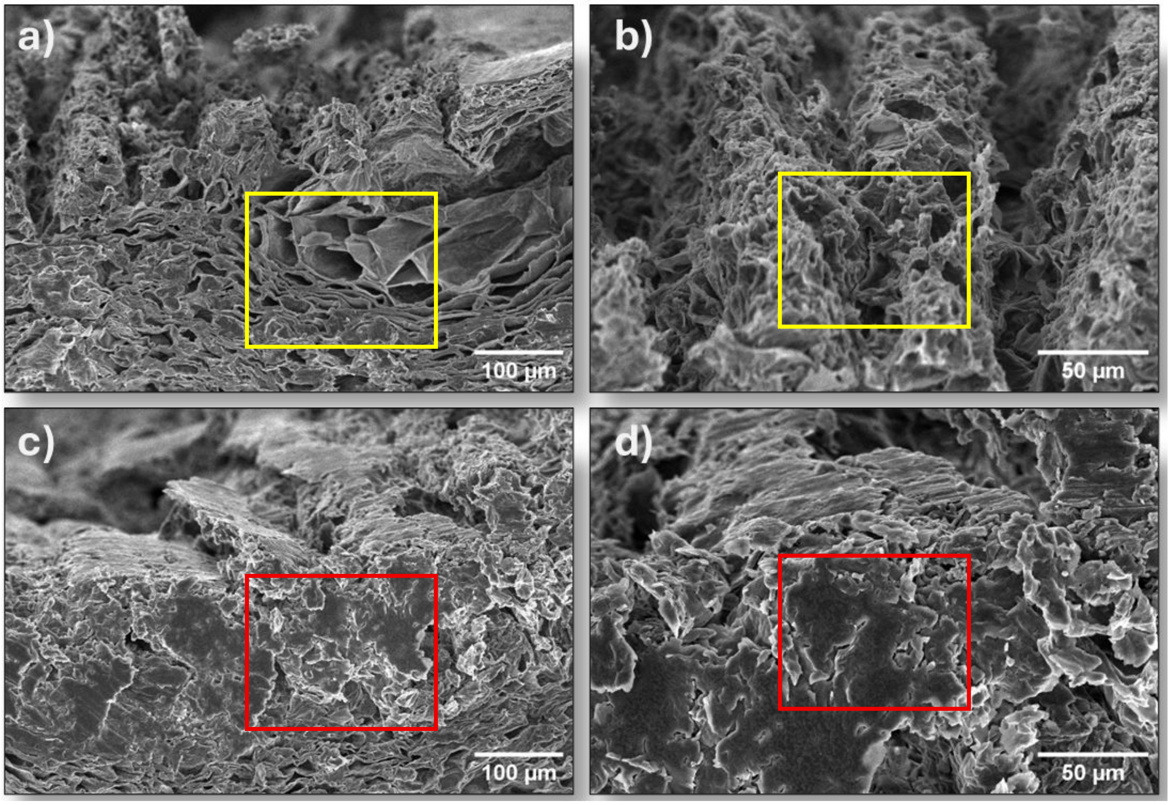

Figure S4: a) Cross-section of OP after LIG without paraffin at 200× magnification; b) 500× magnification, highlighting the pores formed during the carbonization process and the presence of non-carbonized regions; c) LIG with paraffin at 200× magnification; d) 500× magnification, showing the penetration of paraffin into the pores of the sample during the carbonization process. Paraffin infiltration can be observed by comparing the red-highlighted areas in the paraffin-treated sample, where the pores are no longer visible, with those in the untreated sample, in which the porous structure remains clearly exposed.

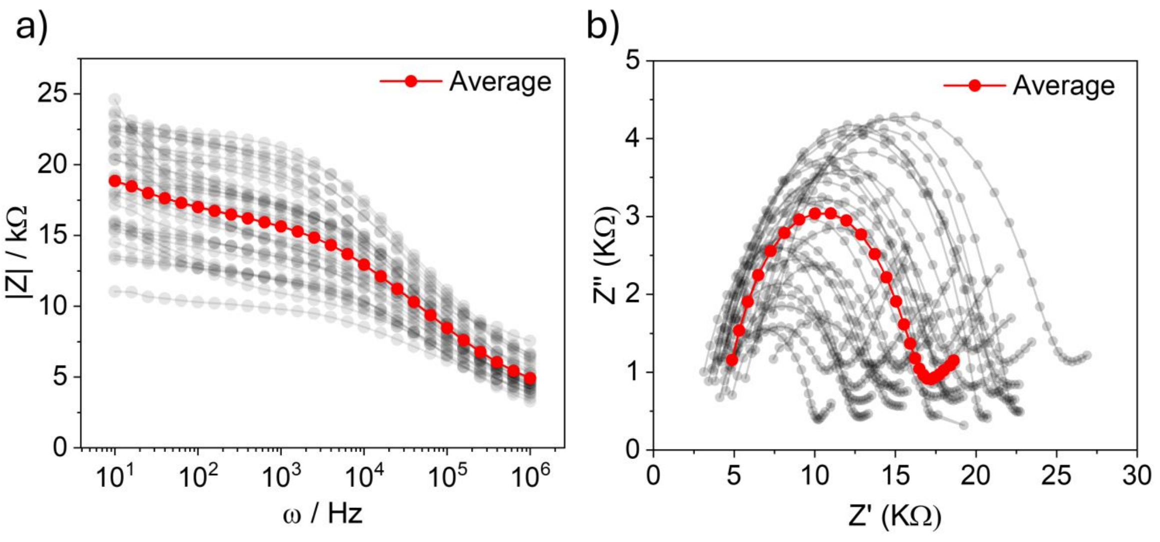

Figure S5: a) Impedance curve of the 30 fabricated electrodes; b) Nyquist plot of the 30 LIG electrodes.
